# Curative effect of anti-fibrosis Chinese patent medicines combined with ursodeoxycholic acid for primary biliary cholangitis: A systematic review and meta-analysis

**DOI:** 10.3389/fphar.2023.1159222

**Published:** 2023-03-21

**Authors:** Yufei Bi, Ke Shi, Jialiang Chen, Xianbo Wang

**Affiliations:** Center of Integrative Medicine, Beijing Ditan Hospital, Capital Medical University, Beijing, China

**Keywords:** primary biliary cholangitis, Chinese patent medicine, anti-fibrosis, ursodeoxycholic acid, meta-analysis

## Abstract

**Objective:** To delineate the curative effect and safety of anti-fibrosis Chinese patent medicines (CPMs) combined with ursodeoxycholic acid (UDCA) for primary biliary cholangitis (PBC).

**Methods:** A literature search was conducted using PubMed, Web of Science, Embase, Cochrane Library, Wanfang database, VIP database, China Biology Medicine Database, and Chinese National Knowledge Infrastructure from their inception until August 2022. Randomized controlled trials (RCTs) of the treatment of PBC with anti-fibrotic CPMs were collected. The eligibility of the publications was assessed using the Cochrane risk-of-bias tool. The evaluation indicators were the clinical efficacy rate, liver fibrosis, liver function, immune function, and symptom score. Meta-analysis and subgroup analysis were conducted to evaluate the effectiveness of anti-fibrosis CPMs. Risk ratio (RR) was used to assess dichotomous variables, and continuous variables with a 95% confidence interval were calculated using mean difference.

**Results:** Twenty-two RCTs including 1,725 patients were selected. The findings demonstrated that anti-fibrotic CPMs combined with UDCA improved the efficacy rate, liver function, liver fibrosis, immunological indicators, and clinical symptoms compared with UDCA alone (all *p* < 0.05).

**Conclusion:** This study demonstrates that the combination of anti-fibrotic CPMs and UDCA can improve both clinical symptoms and outcomes. Nevertheless, more high-quality RCTs are needed to assess the effectiveness of anti-fibrosis CPMs for PBC.

## 1 Introduction

Primary biliary cholangitis (PBC), also called primary biliary cirrhosis, is a chronic autoimmune cholestatic liver disease whose pathogenesis has not been fully elucidated (European Association for the Study of the Liver, 2017). PBC frequently occurs in middle-aged women, and its clinical serological characteristics include positive anti-mitochondrial antibodies and elevated levels of alkaline phosphatase (ALP) or gamma-glutamyl transpeptidase (GGT) (You et al., 2022). The main pathological features of the liver include progressive, non-suppurative, and destructive intrahepatic cholangitis, leading to fibrosis and eventually cirrhosis (Zhang et al., 2021; You et al., 2022; European Association for the Study of the Liver, 2017). PBC is mainly caused by genetic and environmental factors, with unknown pathogenesis and hidden clinical manifestations (You et al., 2022). Some patients with PBC have cirrhosis at the time of diagnosis (Zhang et al., 2021), making anti-fibrotic therapy particularly important.

Ursodeoxycholic acid (UDCA) is an effective treatment for PBC (You et al., 2022), and its mechanism of action includes cholelithiasis, cytoprotection, anti-inflammatory effects, and immune regulation (Zhang et al., 2021). UDCA has greatly improved longevity in patients with PBC. However, 40% of patients with PBC still respond poorly to it (Cheung et al., 2016), and non-responders have a lower survival rate than the general population. Owing to rapid disease progression and poor long-term prognosis, patients who respond poorly to UDCA may require alternative treatment methods urgently; however, currently, no unified therapies exist.

Traditional Chinese medicine (TCM) has advantages in treating liver fibrosis, owing to its combination of ingredients and multiple pathways and targets. Previous studies have shown that Fuzheng Huayu capsules (FZHY) (Liu et al., 2019), Fufang Biejia Ruangan tablets (FFBJRG) (Ji et al., 2022), and Anluo Huaxian pills (ALHX) (Lu et al., 2017) are commonly used clinically as Chinese patent medicines (CPMs) for the anti-fibrosis treatment of liver these have been approved by the State Food and Drug Administration of China, with national medicine permission numbers of Z20020074 (FZHY), Z19991011 (FFBJRG), and Z20010098 (ALHX). The fibrosis stage is important in the progression of PBC. If anti-fibrosis treatment is administered in time at this stage, the occurrence of liver cancer or even liver failure will be reversed (Prince et al., 2002). Additionally, a recent study demonstrated that the treatment of PBC with TCM exerts anti-fibrotic effects and helps improve patients’ pruritus, fatigue, and the response rate to UDCA (Chen et al., 2018). Meanwhile, several studies have revealed that FZHY, FFBJRG, and ALHX have unique advantages in improving biochemical indices, anti-fibrosis, and quality of life in patients with PBC (Chen et al., 2019; Jiang et al., 2019; Wang et al., 2020). Furthermore, a previous real-world cohort study (Chen et al., 2018) conducted by our research group found that TCM combined with UDCA increased the 1-year biochemical response rate of patients with PBC by 15.1% compared with that of UDCA alone (43.0% vs. 27.9%, *p* < 0.05).

Therefore, it is of great significance to explore the clinical curative effects of CPMs combined with UDCA on PBC. Therefore, a meta-analysis of randomized controlled trials (RCTs) was conducted to measure the efficacy of CPMs plus UDCA for treating PBC.

## 2 Materials and methods

### 2.1 Standards for inclusion and exclusion of literature

The inclusion criteria were: 1) the studies reported RCTs; 2) the participants were diagnosed with PBC based on the consensus recommendations of the Asian Pacific Association for the Study of the Liver (APASL); 3) the treatment involved anti-fibrotic CPMs plus UDCA; and 4) at least one of the following outcome indices was used: clinical efficacy rate, ALP, GGT, alanine aminotransferase (ALT), aspartate aminotransferase (AST), hyaluronic acid (HA), laminin (LN), collagen type IV (IV-C), type III procollagen (PC-III), immunoglobulin M (IgM), immunoglobulin G (IgG), and clinical symptoms. The primary outcome was the clinical efficacy rate, whereas the secondary outcomes were liver function, hepatic fibrosis, immunological indicators, and clinical symptoms.

The exclusion criteria were: 1) the experimental group used none of the three aforementioned anti-fibrotic CPMs or other TCMs; 2) duplicate studies; 3) studies with incomplete research data; and 4) animal experiments, conferencing articles, reviews, non-RCTs, and other unrelated studies.

### 2.2 Search strategy

The meta-analysis followed the newest Preferred Reporting Items for Systematic Reviews and Meta-Analyses (PRISMA) statement (Supplementary Table S1) (Page et al., 2021). A literature search was conducted using PubMed, Web of Science, Embase, Cochrane Library, Wanfang database, VIP database, China Biology Medicine Database, and Chinese National Knowledge Infrastructure from their inception until August 2022, with no language restriction. The search terms included: “Primary Biliary Cirrhosis,” “Primary Biliary Cholangitis,” “PBC,” “Chinese patent medicine,” “CPMs,” “traditional Chinese medicine,” “TCM,” “Ursodeoxycholic Acid,” “UDCA,” “Fuzheng Huayu capsules,” “FZHY,” “Fufang Biejia Ruangan tablets,” “FFBJRG,” “Anluo huaxian Pills,” “ALHX,” “ursodeoxycholic acid,” “UDCA,” “randomized controlled trials,” and “RCT.” The search strategy is presented in Table 1.

**TABLE 1:** The search strategy.

Order	Search items
#1	((Primary Biliary Cholangitis [Title/Abstract]) OR (Primary Biliary Cirrhosis [Title/Abstract])) OR (PBC [Title/Abstract])
#2	(ursodeoxycholic acid [Title/Abstract]) OR (UDCA [Title/Abstract])
#3	(((((((((Fuzheng Huayu capsuless [Title/Abstract]) OR (FZHY [Title/Abstract])) OR (Fufang Biejia Ruangan tablets [Title/Abstract])) OR (FFBJRG [Title/Abstract])) OR (Anluo huaxian Pills [Title/Abstract])) OR (ALHX [Title/Abstract])) OR (Chinese patent medicine [Title/Abstract])) OR (CPMs [Title/Abstract])) OR (traditional Chinese medicine [Title/Abstract])) OR (TCM [Title/Abstract])
#4	((randomized controlled trial [Title/Abstract]) OR (RCTs [Title/Abstract])) OR (randomized [Title/Abstract])
#5	#1 AND #2 AND #3AND#4

### 2.3 Data acquisition and quality evaluation

The literature was independently screened by two researchers (BI and SHI) in terms of the inclusion and exclusion standards. Data acquisition consisted of 1) general information: title, first author, and publication year; 2) sex, age, sample size, intervention measures, and treatment course; and 3) observed outcome indicators.

Literature eligibility was estimated using the Cochrane collaboration tool, including incomplete outcome data, selective reporting, allocation concealment, random-sequence generation, blinding of outcome assessment, blinding of participants and personnel, and other biases. Based on these standards, the literature eligibility was categorized as three levels of risk of bias: high, unclear, and low.

### 2.4 Statistical methods

The statistical analysis was performed using RevMan 5.4 software. According to the type of outcome, continuous data are depicted as mean difference (MD) or standardized mean difference (SMD), while categorical data are presented as risk ratios (RRs); all are expressed with a 95% confidence interval (CI). Furthermore, the χ^2^ and *I* ^*2*^ tests were used for heterogeneity analysis. The fixed-effects model was used for analysis if *p* ≥ 0.1 and *I* ^*2*^ ≤ 50% in the subgroup or overall. Conversely, the random-effects model was employed if *p* < 0.1 and *I* ^*2*^ > 50%. Additionally, we searched for sources of heterogeneity in the results and employed a sensitivity analysis to affirm whether the results were stable. Moreover, subgroup analyses were performed based on the use of three different CPMs. A *p*-value less than 0.05 was considered statistically significant. Publication bias was analyzed using funnel plots.

## 3 Results

### 3.1 Study selection

**FIGURE 1:**
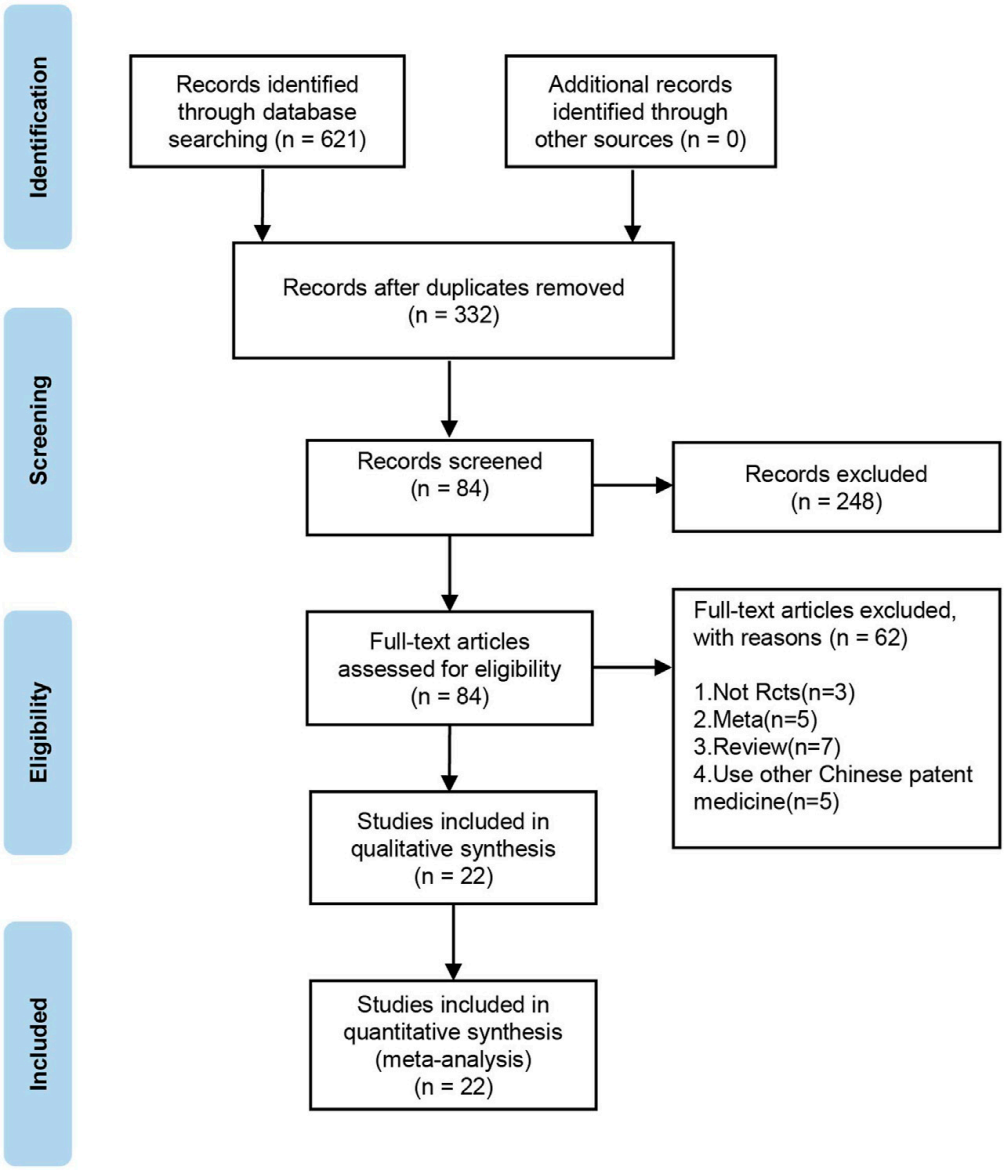
Flow diagram of the literature screening and selection process.

In total, 621 relevant studies were selected in the initial search. After duplicates were removed, 332 studies remained. After screening the titles, abstracts, and introductions of each article, 84 articles were deleted. Finally, after reading the full-text articles for further screening and evaluation, 22 publications (Wu et al., 2012; Xie et al., 2012; Ying, 2012; Li and Wu, 2013; Zhang et al., 2014; Gao et al., 2015; Huang et al., 2015; Qiu and Rao, 2015; Wang, 2015; Wang et al., 2015; Pang, 2017; Yang, 2017; Chen et al., 2019; Jiang et al., 2019; Zhang, 2019; Liu, 2020b; Wang, 2020; Zhang et al., 2020; Ai, 2021; Zhang, 2021) were included. The selection procedure for publications is shown in Figure 1.

### 3.2 Study characteristics

All 22 included studies were RCTs conducted in China and involved 1,725 patients with PBC. All studies were classified into an experimental group and a control group to compare the efficacies of CMPs combined with UDCA and UDCA monotherapy. The basic traits of each study are summarized in Table 2.

**TABLE 2:** Basic characteristics of the included studies.

Included Trial	Sample size	Gender male/female		Age		Intervention		TreatmentDuration	OutcomeIndex
	N1/N2	N1	N2	N1	N2	N1	N2		
Liu 2020a	44/44	16/28	18/26	55.28 ± 6.28	55.19 ± 6.32	FZHY + UDCA	UDCA	48 W	2)7)
Jiang et al., 2019	41/41	4/37	5/36	N	N	FZHY + UDCA	UDCA	12 M	1)2)8)
Yang 2017	60/60	15/45	13/47	52.5 ± 7.4	51.6 ± 6.8	FZHY + UDCA	UDCA	12 W	1)2)
Gao et al. (2015)	31/30	2/29	2/28	43.60 ± 10.4	45.20 ± 9.6	FZHY + UDCA	UDCA	24 W	1)2)3)8)
Zhang et al. (2014)	30/30	3/27	3/27	56.52 ± 10.42	58.04 ± 9.80	FZHY + UDCA	UDCA	24 W	2)5)7)
Wu et al. (2012)	40/40	5/35	4/36	43.7 ± 12.0	45.0 ± 11.6	FZHY + UDCA	UDCA	48 W	1)2)3)5)6)7)8)
Ying 2012	65/65	19	111	N	N	FZHY + UDCA	UDCA	24 W	1)2)8)
Xie et al. (2012)	15/14	3	26	N	N	FZHY + UDCA	UDCA	6 M	1)3)5)
Zhang 2021	48/47	11/37	8/39	48.46 ± 5.66	47.91 ± 4.86	FFBJRG + UDCA	UDCA	6 M	3)4)8)
Ai 2021	43/43	5/38	4/39	42.69 ± 3.12	42.57 ± 3.09	FFBJRG + UDCA	UDCA	6 M	3)7)
Liu 2020b	38/38	6/32	5/33	35.65 ± 3.52	36.58 ± 4.21	FFBJRG + UDCA	UDCA	6 M	2)
Wang 2020	36/36	16/20	17/19	41.21 ± 2.37	41.56 ± 2.25	FFBJRG + UDCA	UDCA	6 M	2)
Zhang et al. (2020)	21/21	N	N	N	N	FFBJRG + UDCA	UDCA	6 M	1)2)3)5)8)
Zhang 2019	85/85	17/68	21/64	43.9 ± 13.6	42.7 ± 14.1	FFBJRG + UDCA	UDCA	6 M	1)5)7)
Chen et al. (2019)	41/37	N	N	N	N	FFBJRG + UDCA	UDCA	24 W	1)3)5)
Pang 2017	25/25	9/16	10/15	52.1 ± 6.7	52.3 ± 7.1	FFBJRG + UDCA	UDCA	24 M	1)2)
Zhang et al. (2015)	32/32	4	60	N	N	FFBJRG + UDCA	UDCA	12 M	1)2)3)
Wang 2015	39/39	8/31	6/33	53.7 ± 6.9	52.1 ± 7.4	FFBJRG + UDCA	UDCA	12 M	2)4)
Huang et al. (2015)	28/28	3/25	4/24	57.97 ± 9.07	57.59 ± 8.46	FFBJRG + UDCA	UDCA	12 M	1)4)5)
Qiu and Rao 2015	42/42	15/27	12/30	31.6 ± 1.9	31.2 ± 1.5	ALHX + UDCA	UDCA	24 W	1)2)3)5)6)7)8)
Wang et al. (2015)	32/32	5/27	6/26	47.5	48.2	ALHX + UDCA	UDCA	3 M	1)2)3)
Li and Wu 2013	30/30	2/28	3/27	45.6 ± 10.3	42.2 ± 9.2	ALHX + UDCA	UDCA	24 W	1)2)3)

Abbreviations: N1, experimental group; N2, control group; W, weeks; M, months; FZHY, fuzheng huayu capsules; FFBJRG, fufang biejia ruangan tablets; ALHX, anluo huaxian pills; Outcome Index: 1) clinical efficacy rate; 2) liver function; 3) hepatic fibrosis; 4) acoustic radiation force impulse imaging; 5) symptoms; 6) portal dynamics; 7) immune function; 8) adverse effects.

### 3.3 Quality assessment

**FIGURE 2:**
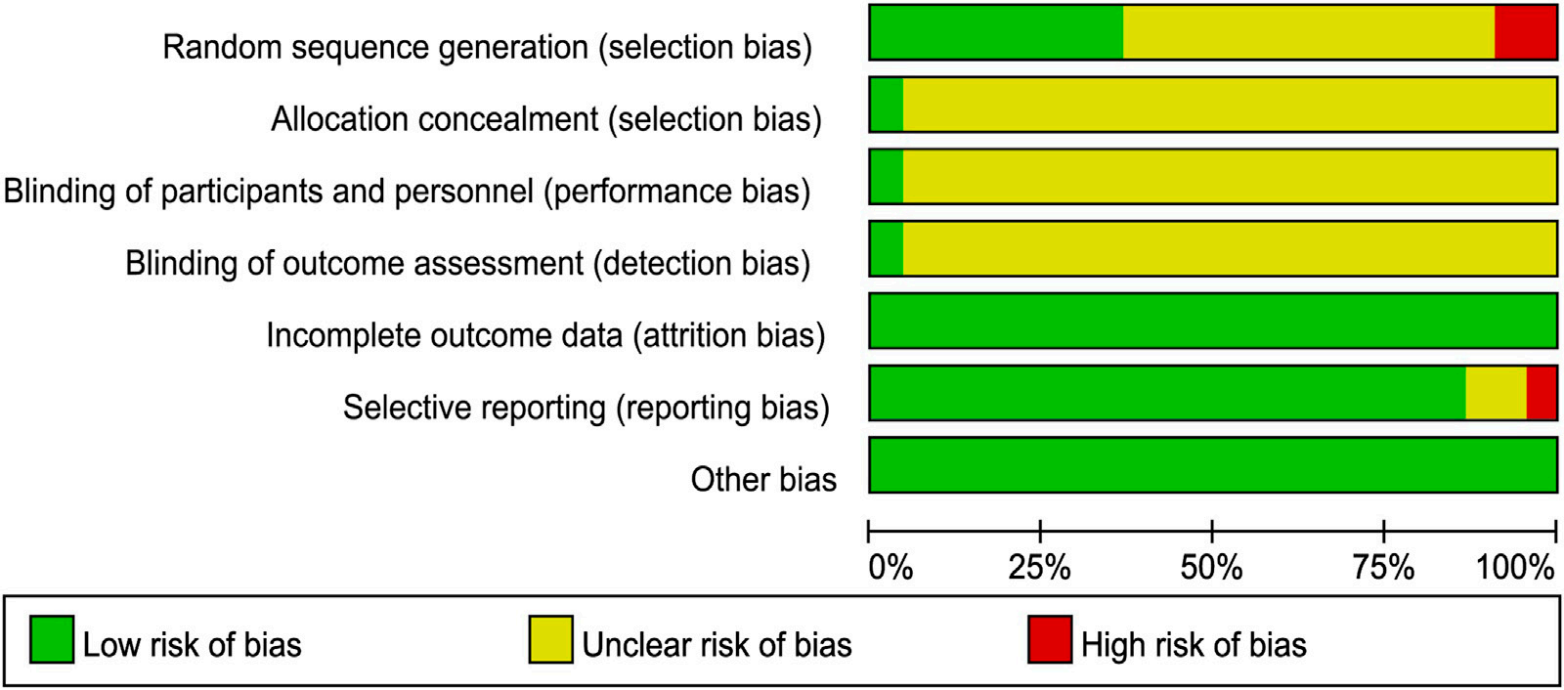
Risk of bias graph.

**FIGURE 3:**
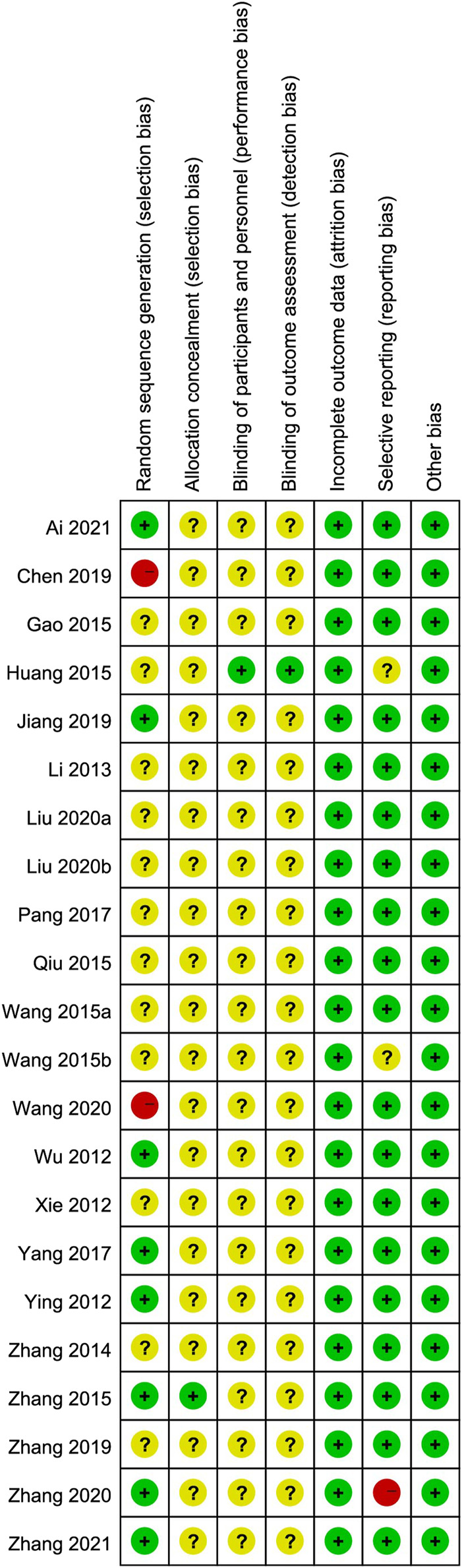
Summary of risk of bias assessment.

We used the Cochrane risk-of-bias tool to estimate article quality. Of these, eight publications (Wu et al., 2012; Ying, 2012; Zhang et al., 2015; Yang, 2017; Jiang et al., 2019; Zhang et al., 2020; Ai, 2021; Zhang, 2021) used a random number table, 12 (Xie et al., 2012; Li and Wu, 2013; Zhang et al., 2014; Huang et al., 2015; Qiu and Rao, 2015; Wang, 2015; Wang et al., 2015; Cheung et al., 2016; Pang, 2017; Zhang, 2019; Liu, 2020a; Liu, 2020b) did not describe the randomization method, and two (Wang 2020; Chen et al., 2019 did not mention the word “random.” Only one publication (Zhang et al., 2015) used a double-blind design and reported the number of missing cases in the experimental and control groups. In terms of hidden allocation, one publication (Huang et al., 2015) adopted a non-transparent envelope. The details of the risk of bias assessment are shown in Figures 2, 3.

### 3.4 CPMs drug composition

These studies used three anti-fibrosis CPMs, including FZHY, FFBJRG, and ALHX, and listed the TCMs used. Dongchongxiacao (*Cordyceps sinensis* (Berk.) Sacc) is an ingredient in FZHY and FFBJRG, while Sanqi (*Panax notoginseng* (Burkill) F. H. Chen) is an ingredient in ALHX and FFBJRG. The compositions of the anti-hepatic fibrosis herbal formulations are listed in Table 3.

**TABLE 3:** The herbal formula of FZHY, FFBJRRG and ALHX.

Herbal formula	Chinese name	Latin name	Picture
FZHY	Danshen	*Salvia miltiorrhiza Bge*	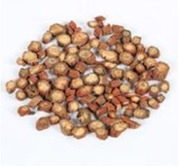
	Dongchongxiacao	*Cordyceps sinensis (Berk.) Sacc*	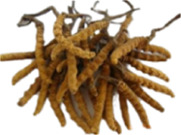
	Taoren	*Prunus persica* (*L.*) *Batsch*	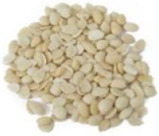
	Jiaogulan	*Gynostemma aggregatum C.Y.Wu and S.K.Chen*	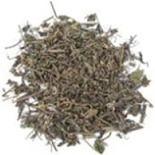
	Songhuafen	*Pollen Pini*	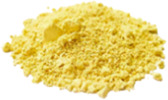
	Wuweizi	Schisandra chinensis (Turcz.) *Baill*	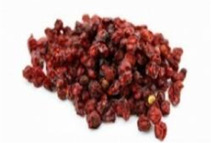
FFBJRG	Biejia	Shell of *Trinyx sinensis Widgmann*	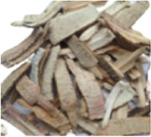
	Danggui	*Angelica sinensis (Oliv.) Diels*	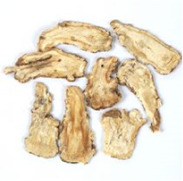
	Dangshen	*Codonopsis pilosula (Franch.) Nannf*	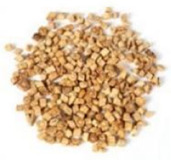
	Chishao	*Paeonia Lactiflora Pall*	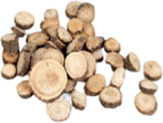
	Ezhu	*Rhizoma curcumae*	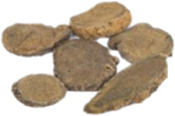
	Sanqi	*Panax notoginseng (Burk.) F. H. Chen*	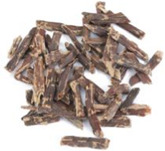
	Ziheche	*Placenta hominis*	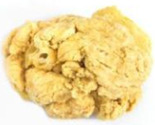
	Dongchongxiacao	*Cordyceps sinensis (Berk.) Sacc*	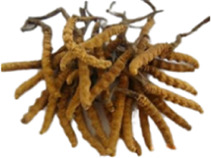
	Huangqi	*Astragali Radix*	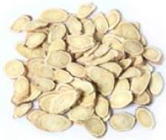
	Banlangen	*Isatis indigotica Fortune ex Lindl*	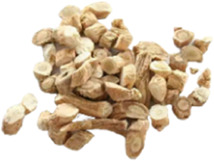
	Lianqiao	*Forsythia suspensa* (Thunb.) Vahl	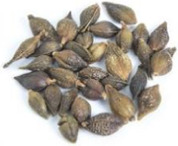
ALHX	Sanqi	*Panax notoginseng (Burk.) F. H. Chen*	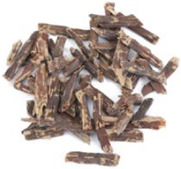
	Shuizhi	*Whitmania pigra Whitman*	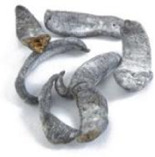
	Shengdihuang	*Rehmannia glutinosa Libosch*	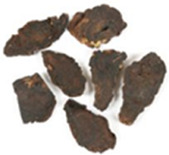
	Jiangcan	*Bombyx mori Linnaeus*	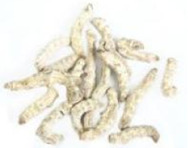
	Baizhu	*Atractylodes macrocephala Koidz*	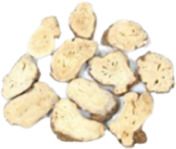
	Dilong	*Pheretima aspergillum (E. Perrier)*	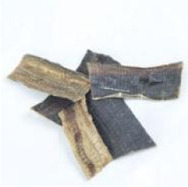
	Yujin	*Curcuma wenyujin Y.H.Chen and C.Ling*	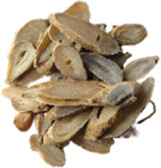
	Niuhuang	*Bezoar*	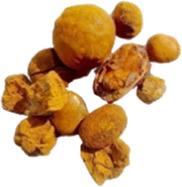
	Walengzi	*Arca subcrenata Lischke*	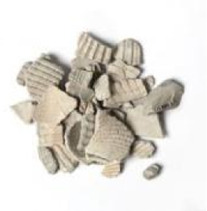
	Mudanpi	*Paeonia suffruticosa Andr*	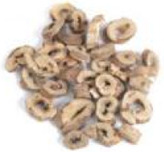
	Dahuang	*Rheum officinale Baill*	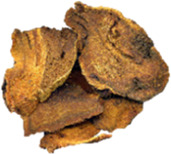
	Shengmaiya	*Hordeum vulgare L*	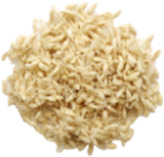
	Shuiniujiao	*horn of Bubalus bubalis Linnaeus*	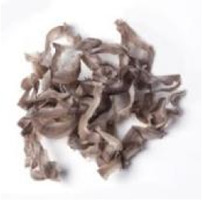
	Jineijin	*Endothelium Corneum Gigeriae Galli*	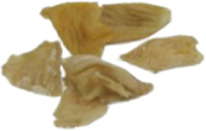

### 3.5 Outcome index

#### 3.5.1 Clinical efficacy rate

**FIGURE 4:**
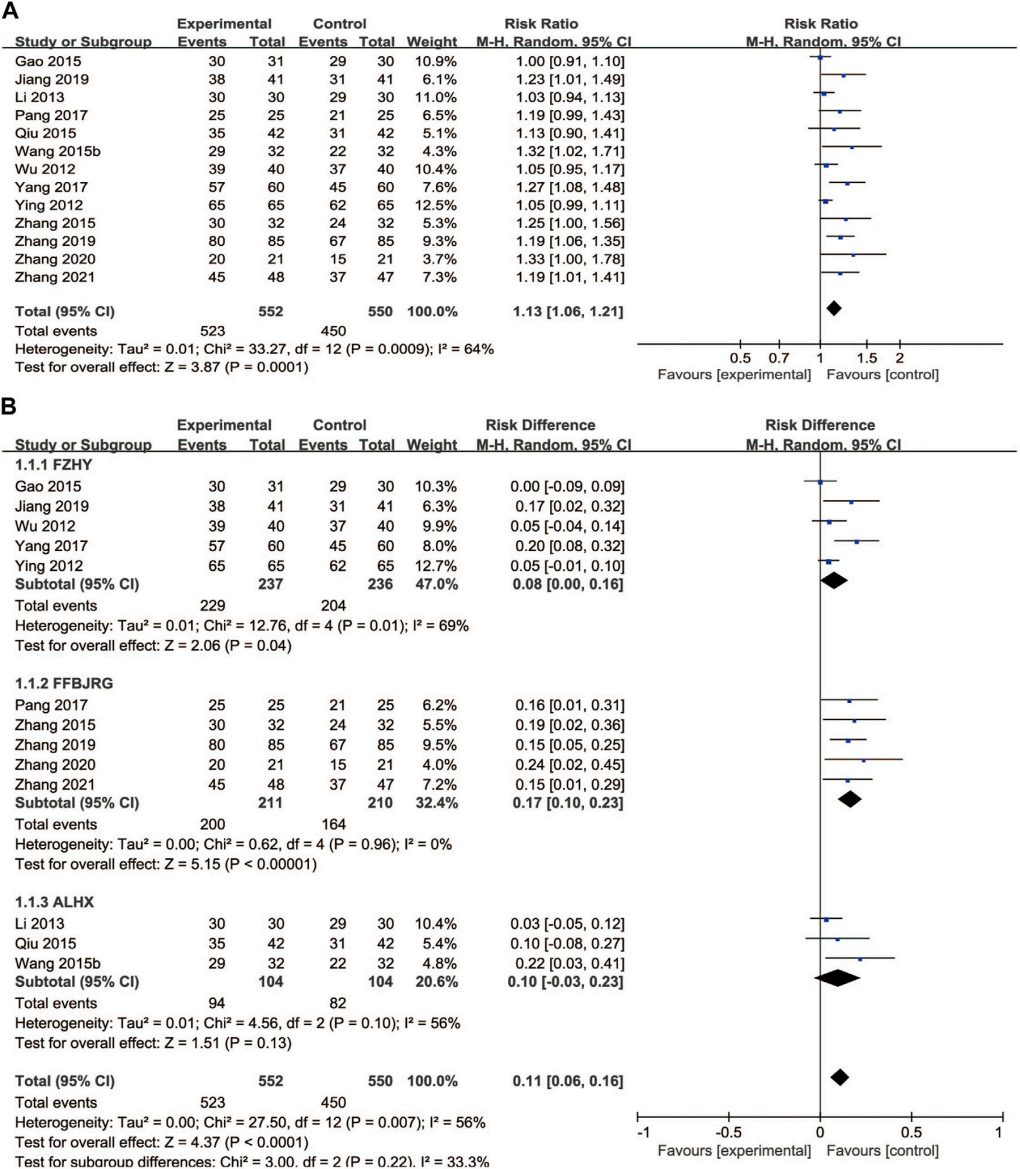
Forest plot of the meta-analysis **(A)** and subgroup analysis **(B)** of clinical efficacy rate.

A total of 13 clinical trials described the clinical efficacy rate as the main outcome, which was classified as markedly effective, effective, and ineffective grades. A random-effects model was implemented for analysis regarding heterogeneity testing (*p* = 0.0009, *I* ^*2*^ = 64%), and anti-fibrosis CPMs plus UDCA improved the clinical efficacy rate compared with UDCA alone (RR = 0.11, 95% CI: 0.06, 0.16; *p* < 0.00001) (Figure 4A). Subgroup analysis showed that the clinical efficacy rate of FFBJRG was better than that of the other two CPMs and had no heterogeneity (*p* = 0.96, *I* ^*2*^ = 0%) (Figure 4B).

### 3.6 Liver function

#### 3.6.1 Alkaline phosphatase

**FIGURE 5:**
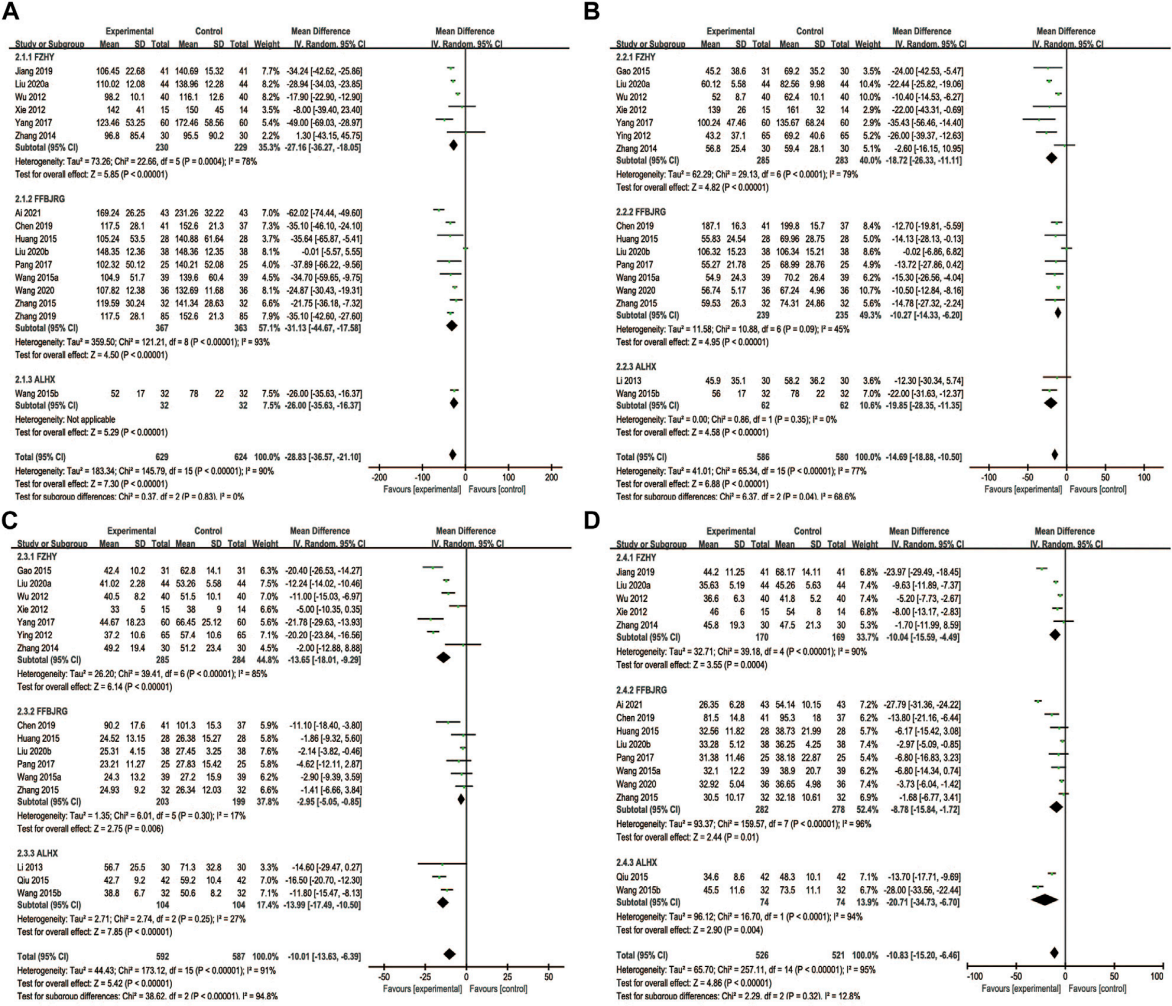
Forest plot of meta-analysis of liver function **(A)** alkaline phosphatase; **(B)** gamma-glutamyl transpeptidase **(C)** alanine aminotransferase; and **(D)** aspartate aminotransferase.

We found 10 trials that reported the effects of anti-fibrotic CPMs plus UDCA on ALP levels. A random-effects model was implemented based on the *p*-value and *I* ^*2*^ value. The subgroup analyses showed that anti-fibrotic CPMs plus UDCA were superior to UDCA alone in terms of serum ALP levels (RR = −28.83, 95% CI: −36.57, −21.10; *p* < 0.00001) (Figure 5A).

#### 3.6.2 Gamma-glutamyl transpeptidase

A total of 16 articles reported GGT levels as the outcome. In the subgroup analyses, random-effects models were used due to heterogeneity (*p* < 0.00001, *I* ^2^ = 77%). Consequently, the anti-fibrotic CPMs plus UDCA were more effective at decreasing GGT levels than UDCA alone (MD = −14.69, 95% CI: −18.88, −10.50; *p* < 0.00001) (Figure 5B).

#### 3.6.3 Alanine aminotransferase

Sixteen studies described ALT levels as the main outcome. A random-effects model was implemented for the analysis based on the heterogeneity test (*p* < 0.00001, *I* ^*2*^ = 91%). Overall, ALT levels significantly decreased after the combination of anti-fibrotic CPMs and UDCA (MD = −10.01, 95% CI: −13.63, −6.39; *p* < 0.00001) (Figure 5C **)**.

#### 3.6.4 Aspartate aminotransferase

Sixteen studies reported serum AST levels as the main outcome. The AST data were processed using a random-effects model because AST was heterogeneous (*p* < 0.00001, *I* ^*2*^ = 95%). The use of anti-fibrotic CMPs plus UDCA showed a greater decrease in serum AST levels than that with UDCA alone (MD = −10.83, 95% CI: −15.20, −6.46; *p* < 0.00001) (Figure 5D **)**.

### 3.7 Liver fibrosis

**FIGURE 6:**
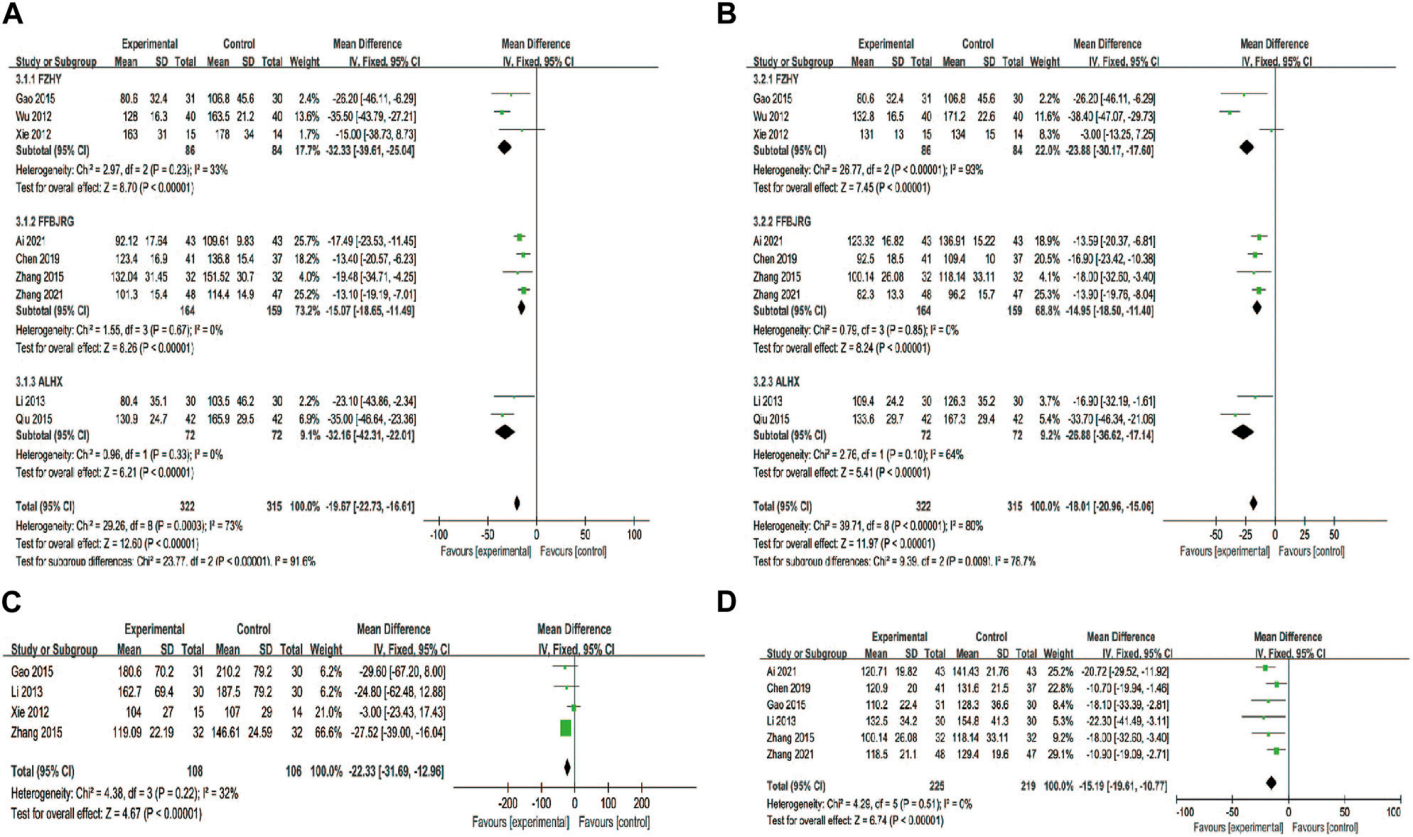
Forest plot of meta-analysis of liver fibrosis **(A)** laminin; **(B)** type IV collagen **(C)** hyaluronic acid; and **(D)** type III procollagen.

Nine trials reported the serum LN and IV-C levels, and we performed a subgroup analysis on these. The findings revealed that the LN (MD = −19.67, 95% CI: −22.73, −16.61) and IV-C (MD = −18.01, 95% CI: −20.96, −15.06) notably decreased after treatment with the combination of anti-fibrotic CMPs plus UDCA (*p* < 0.00001) (Figures 6A, B).

HA levels were reported in four clinical studies, and PC-III was reported as an outcome indicator in six. After testing for heterogeneity, HA was analyzed using a fixed-effects model (*p* = 0.22, *I* ^*2*^ = 32%) and PC-III (*p* = 0.51, *I* ^*2*^ = 0) levels. The experimental group showed a greater decrease in serum HA (MD = −22.33, 95% CI: −31.69, −12.96) and PC-Ⅲ (MD = −15.19, 95% CI: −19.61, −10.77) levels than the control group (*p* < 0.00001) (Figures 6C, D).

### 3.8 Immune function

#### 3.8.1 Immunoglobulin M and immunoglobulin G

**FIGURE 7:**
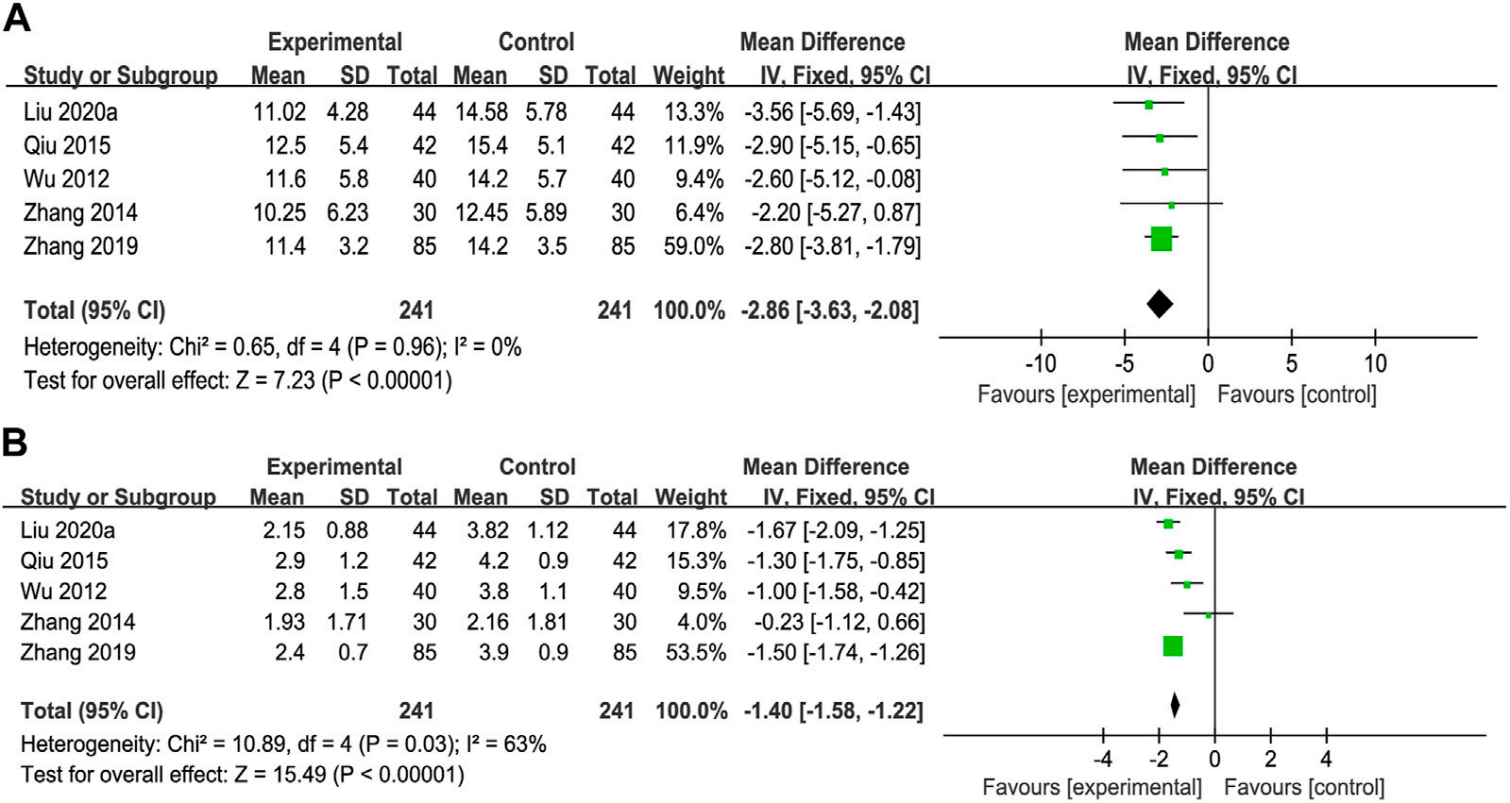
Forest plot of meta-analysis of immune function **(A)** immunoglobulin G (IgG); and **(B)** immunoglobulin M (IgM).

The IgG data were processed using a fixed-effects model based on the heterogeneity test (*p* = 0.96, *I* ^*2*^ = 0%), and a random-effects model was used for the IgM data with high heterogeneity (*p* = 0.03, *I* ^*2*^ = 63%). The MD (95% CI) of IgG and IgM were −2.86 (−3.63, −2.08) and −1.28 (−1.62, −0.94), respectively. Compared with UDCA alone, the additional use of anti-fibrosis CPMs was more effective in reducing IgG and IgM levels **(** Figures 7A, B).

### 3.9 Symptom score

**FIGURE 8:**
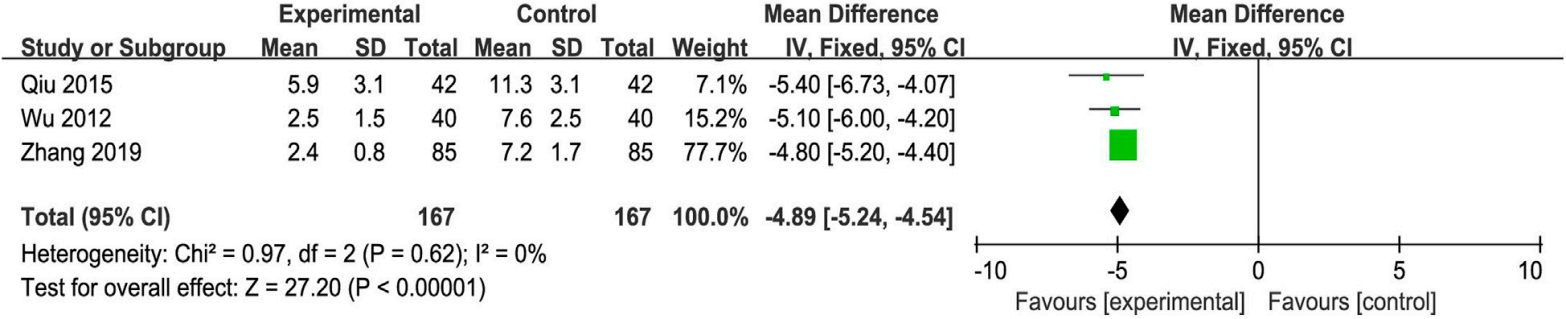
Forest plot of meta-analysis of syndrome score.

Three trials reported Symptom Score Change as the main outcome. The fixed-effects model was adopted because of the absence of heterogeneity (*p* = 0.62, *I* ^*2*^ = 0%). The improvement in the Symptom Score Change was better in the experimental group than in the control group (MD = −4.89, 95% CI: −5.24, −4.54; *p* < 0.00001) (Figure 8).

### 3.10 Adverse events

Adverse events were described in seven studies. Three articles reported a total of nine patients who developed mild diarrhea and nausea after taking CPM; however, these symptoms were not severe and did not require treatment. Four studies reported no adverse reactions.

### 3.11 Publication bias

**FIGURE 9:**
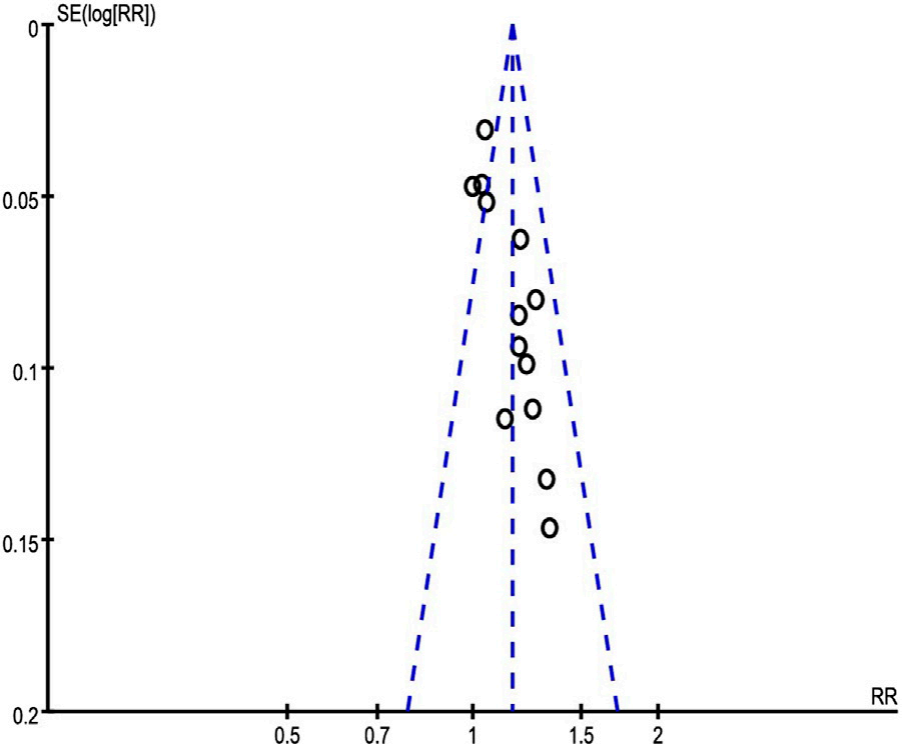
The funnel plot of total effective rate.

An inverted funnel plot analysis of the clinical efficacy rate revealed that there may be publication bias in the asymmetric distribution of these publications (Figure 9).

## 4 Discussion

PBC is the most common autoimmune cirrhotic hepatic disease, occurring in all ethnic groups worldwide (Lv et al., 2020). The prognosis of patients with PBC mostly depends on the degree of liver fibrosis and its complications (Lammers et al., 2015). Patients with PBC tend to seek additional pharmacological treatments because UDCA is not uniformly effective (Lammers et al., 2015). In recent years, TCM has been widely studied and discussed as a complementary therapy. Many studies have shown that TCM plus UDCA has diverse advantages in relieving the clinical symptoms and improving the prognosis of PBC.

This meta-analysis validated the advantages of FZHY, FFBJRG, and ALHX combined with UDCA in the treatment of PBC. Compared with UDCA treatment alone, anti-fibrotic CPMs plus UDCA improved efficacy rates. Furthermore, liver function tests are widely used in clinical practice as indicators of the degree of liver damage. ALP, ALT, AST, and GGT levels decreased after combined treatment with anti-fibrotic CPMs and UDCA. LN, IV-C, PC-III, and HA are indicators for the detection of liver fibrosis, and the addition of anti-fibrosis CPMs to UDCA resulted in decreased levels of these compared with UDCA treatment alone. Moreover, immunological indicators (IgM and IgG) and clinical symptoms also notably improved with the combined treatment of anti-fibrotic CPMs and UDCA. In conclusion, anti-fibrotic CPMs combined with UDCA in the treatment of PBC effectively relieved various clinical indicators. These results provide hope for the treatment and prevention of liver fibrosis and cirrhosis in the future.

The pathobiology of PBC is characterized by inflammation, bile duct damage, and fibrosis (You et al., 2022), of which fibrosis appears in stage Ⅱ. It is believed that TCM is hepatoprotective and anti-inflammatory and suppresses the activation of hepatic stellate cells, which is advantageous in the treatment of liver fibrosis. Additionally, TCM may have multi-level, multi-pathway, and multi-target pharmacological actions on the comprehensive pathogenesis of PBC. For example, *P. notoginseng* is an ingredient in FFBJRG and ALHX, and *P. notoginseng* saponins are its main active constituent, which play an immunomodulatory role by reducing the levels of pro-inflammatory cytokines (Jiang et al., 2013). Furthermore, *Ophiacordyceps sinensis*, as a duplicate herb in FZHY and FFBJRG, can attenuate liver inflammation and fibrosis by regulating the expression of the TGF-β/MAPK pathway (Fu et al., 2021). Moreover, a mechanistic study has revealed that FZHY can decrease the expression levels of α-SMA, CTGF, TIMP-1, TGF-β1, and Smads, thereby reducing hepatic apoptosis, acute liver injury, and liver fibrosis (Cheng et al., 2013; Xie et al., 2013). An animal experiment reported that FFBJRG ameliorates hepatic disease by reducing the serum collagen levels of LN, HA, and IV-C and downregulating TGF-β-Smad pathway fibroblast signal transduction (Yang et al., 2013). The possible mechanism of ALHX for the inhibition of hepatic fibrosis is related to enhancing MMP2 activity in liver tissue and promoting extracellular matrix degradation by hepatoprotective enzymes (Tan et al., 2010). In brief, certain evidence support the anti-inflammatory and anti-fibrosis effects of FZHY, FFBJRG, and ALHX as the appropriate CPMs for treating PBC.

However, this meta-analysis had some limitations. First, the 22 studies included had a small sample size, all were Chinese, and only a small number of studies reported several outcome indicators. Second, most publications only mentioned random assignment, and only one-third of the studies described a specific randomization method, such as a random number table. Therefore, the findings need to be further evaluated using high-quality trials. Third, different studies had different experimental periods, ranging between 12 and 48 weeks, which may be a source of heterogeneity. Fourth, although anti-fibrosis CPMs were always used in the experimental group, there were three different types—FZHY, FFBJRG, and ALHX, which could also be a source of heterogeneity. Finally, because half of the studies did not mention adverse events, the safety of CPMs as an anti-fibrosis therapy for PBC should be further evaluated, and caution is needed when drawing conclusions.

## 5 Conclusion

Our research shows that the combination of anti-fibrosis CPMs and UDCA is more effective than UDCA alone in treating PBC in improving clinical efficacy rate, liver fibrosis, liver function, immune function, and symptom score. This systematic review and meta-analysis provides reliable clinical evidence for PBC treatment. Anti-fibrotic CPMs are a promising therapeutic approach to supplement the conventional treatment of PBC. However, further evidence from high-quality and multi-center studies with larger samples is warranted to confirm the curative effect of anti-fibrosis CPMs during follow-up periods.

## Data Availability

The original contributions presented in the study are included in the article/Supplementary Materials, further inquiries can be directed to the corresponding author.

## Abbreviations

ALHX, anluo huaxian pills; ALP, alkaline phosphatase; ALT, alanine aminotransferase; AST, aspartate aminotransferase; APASL, asian pacific association for the study of the liver; CI, confidence interval; CPM, Chinese patent medicine; FDA, United States Food and Drug Administration; FFBJRG, Fufang Biejia Ruangan tablets; FZHY, Fuzheng Huayu capsules; GGT, gamma-glutamyl transpeptidase; HA, hyaluronidase; IgG, immunoglobulin G; IgM, immunoglobulin M; IV-C, type IV collagen; LN, laminin; MD, mean difference; PBC, primary biliary cholangitis; PC-III, type III procollagen; PRISMA, Preferred Reporting Items for Systematic Reviews and Meta-Analyses; RCTs, randomized controlled trials; RR, risk ratio; SMD, standardized mean difference; TCM, traditional Chinese medicine; UDCA, ursodeoxycholic acid.
