# Supplementary material for: Curative effect of anti-fibrosis Chinese patent medicines combined with ursodeoxycholic acid for primary biliary cholangitis: A systematic review and meta-analysis
Source: Front Pharmacol. 2023 Mar 21;14:1159222. doi: 10.3389/fphar.2023.1159222 (PMC10070848; doi:10.3389/fphar.2023.1159222)
Supplement: Supplementary file 1 [file Table1.DOC]

| **Section and topic** | **Item#** | **Checklist item** | **Location where item is reported** |
| --- | --- | --- | --- |
| **TITLE** | | |  |
| Title | 1 | Identify the report as a systematic review and meta-analysis | line 1-3，  page 1 |
| **ABSTRACT** | | |  |
| Abstract | 2 | The structured abstract includes: objectives; data sources; study eligibility criteria, participants, and interventions; study appraisal and synthesis methods; results; limitations; conclusions and implications of key findings; systematic review registration number. | line13-33,  page 1-2 |
| **INTRODUCTION** | | |  |
| Rationale | 3 | Describe the rationale for the review in the context of existing knowledge. | line 36-57, page 2-3 |
| Objectives | 4 | Provide an explicit statement of the objective(s) or question(s) the review addresses. | line 75-75,  page 4-5 |
| **METHODS** | | |  |
| Eligibility criteria | 5 | Specify the inclusion and exclusion criteria for the review and how studies were grouped for the syntheses. | line 82-95, page 4 |
| Information sources | 6 | Specify all databases, registers, websites, organisations, reference lists and other sources searched or consulted to identify studies. Specify the date when each source was last searched or consulted. | line 102-107，  page 5 |
| Search strategy | 7 | Present the full search strategies for all databases, registers and websites, including any filters and limits used. | line 97-117，  page 5-6 |
| Selection process | 8 | Specify the methods used to decide whether a study met the inclusion criteria of the review, including how many reviewers screened each record and each report retrieved, whether they worked independently, and if applicable, details of automation tools used in the process. | line 109-110  page 5 |
| Data collection process | 9 | Describe method of data extraction from reports (e.g., piloted forms, independently, in duplicate) and any processes for obtaining and confirming data from investigators. | line 114-116，  page 5 |
| Data items | 10a | List and define all outcomes for which data were sought. Specify whether all results that were compatible with each outcome domain in each study were sought (e.g. for all measures, time points, analyses), and if not, the methods used to decide which results to collect. | line 85-89，  page 4-5 |
|  | 10b | \| List and define all other variables for which data were sought (e.g. participant and intervention characteristics, funding sources). Describe any assumptions made about any missing or unclear information. \| \| --- \| | line 110-112，page 5-6 |
| Study risk of bias assessment | 11 | Specify the methods used to assess risk of bias in the included studies, including details of the tool(s) used, how many reviewers assessed each study and whether they worked independently, and if applicable, details of automation tools used in the process. | line 113-117，  page 6 |
| Effect measures | 12 | Specify for each outcome the effect measure(s) (e.g. risk ratio, mean difference) used in the synthesis or presentation of results. | line 119-121, page 6 |
| Synthesis methods | 13a | Describe the processes used to decide which studies were eligible for each synthesis (e.g. tabulating the study intervention characteristics and comparing against the planned groups for each synthesis (item #5)). | line 132-141, page 7 |
|  | 13b | Describe any methods required to prepare the data for presentation or synthesis, such as handling of missing summary statistics, or data conversions. | line 132-141, page 7 |
|  | 13c | Describe any methods used to tabulate or visually display results of individual studies and syntheses. | line 143-141  page 7, table 1 |
|  | 13d | Describe any methods used to synthesise results and provide a rationale for the choice(s). If meta-analysis was performed, describe the model(s), method(s) to identify the presence and extent of statistical heterogeneity, and software package(s) used. | line 123-125，  page 6 |
|  | 13e | Describe any methods used to explore possible causes of heterogeneity among study results (e.g. subgroup analysis, meta-regression). | line 125-127，page 6 |
|  | 13f | Describe any sensitivity analyses conducted to assess robustness of the synthesised results. | line122-123，page 6 |
| Reporting bias assessment | 14 | Describe any methods used to assess risk of bias due to missing results in a synthesis (arising from reporting biases). | line 128-129, page 6 |
| Certainty assessment | 15 | Describe any methods used to assess certainty (or confidence) in the body of evidence for an outcome. | line 122，  page 6 |

Page 1 of 2

| **Section/topic** | **#** | **Checklist item** | **Reported on page #** |
| --- | --- | --- | --- |
| **RESULTS** | | |  |
| Study selection | 16a | Describe the results of the search and selection process, from the number of records identified in the search to the number of studies included in the review, ideally using a flow diagram | line 133-141，page 7，figure1 |
|  | 16b | Cite studies that might appear to meet the inclusion criteria, but which were excluded, and explain why they were excluded. | line 135-141，page 7，figure1 |
| Study characteristics | 17 | For each study, present characteristics for which data were extracted (e.g., study size, PICOS, follow-up period) and provide the citations. | line 143-146，page 7, table 1 |
| Risk of bias in studies | 18 | Present assessments of risk of bias for each included study. | line 148-158，page 7-8 |
| Results of individual studies | 19 | For all outcomes, present, for each study: (a) summary statistics for each group (where appropriate) and (b) an effect estimate and its precision (e.g. confidence/credible interval), ideally using structured tables or plots. | lIne 147-152，page7，table1 |
| Results of syntheses | 20a | For each synthesis, briefly summarise the characteristics and risk of bias among contributing studies. | line 165-222，page 8-11 |
|  | 20b | Present results of all statistical syntheses conducted. If meta-analysis was done, present for each the summary estimate and its precision (e.g. confidence/credible interval) and measures of statistical heterogeneity. If comparing groups, describe the direction of the effect. | line 165-222，page 8-11 |
|  | 20c | Present results of all investigations of possible causes of heterogeneity among study results. | line 165-222, page 8-11 |
|  | 20d | Present results of all sensitivity analyses conducted to assess the robustness of the synthesised results. | line 165-222，page 8-11 |
| Reporting biases | 21 | Present assessments of risk of bias due to missing results (arising from reporting biases) for each synthesis assessed. | line 228-230，  page 11 |
| Certainty of evidence | 22 | Present assessments of certainty (or confidence) in the body of evidence for each outcome assessed. | line 165-222，page 8-11 |
| **DISCUSSION** | | |  |
| Summary of evidence | 23a | Provide a general interpretation of the results in the context of other evidence. | line 241-253，page 11-12 |
|  | 23b | Discuss any limitations of the evidence included in the review. | line 275-286，page 13 |
|  | 23c | Discuss any limitations of the review processes used. | line 275-286，page 13 |
|  | 23d | Discuss implications of the results for practice, policy, and future research. | line 291-296，page 14 |
| **OTHER INFORMATION** | | |  |
| Registration and protocol | 24 | Provide registration information for the review, including register name and registration number, or state that the review was not registered. Indicate where the review protocol can be accessed, or state that a protocol was not prepared. Describe and explain any amendments to information provided at registration or in the protocol. | No |
| Support | 25 | Describe sources of financial or non-financial support for the review, and the role of the funders or sponsors in the review. | line 303-304，page 14 |
| Competing interests | 26 | Declare any competing interests of review authors. | line 305-307，page 14-15 |
| Availability of data, code, and other materials | 27 | Report which of the following are publicly available and where they can be found: template data collection forms; data extracted from included studies; data used for all analyses; analytic code; any other materials used in the review. | No |
